# Prenatal Exposure to Perfluoroalkyl Substances and Birth Outcomes; An Updated Analysis from the Danish National Birth Cohort

**DOI:** 10.3390/ijerph15091832

**Published:** 2018-08-24

**Authors:** Qi Meng, Kosuke Inoue, Beate Ritz, Jørn Olsen, Zeyan Liew

**Affiliations:** 1 Department of Epidemiology, Fielding School of Public Health, University of California, Los Angeles (UCLA), Los Angeles, CA 90095, USA; quinn7meng@gmail.com (Q.M.); koinoue@ucla.edu (K.I.); britz@ucla.edu (B.R.); 2 Department of Clinical Epidemiology, Aarhus University Hospital, N 8200 Aarhus, Denmark; jo@ph.au.dk

**Keywords:** Danish National Birth Cohort, perfluoroalkyl substances, endocrine disrupters, pregnancy, fetal growth, birth outcomes

## Abstract

Perfluoroalkyl substances (PFASs) are widespread industrial pollutants that are extremely persistent in the environment. A previous study in the Danish National Birth Cohort (DNBC) found prenatal perfluorooctanoate (PFOA) exposure was associated with decreased birth weight, but had insufficient statistical power to evaluate adverse birth outcomes. Here, we conducted additional analyses in three samples originating from the DNBC for 3535 mothers and infant pairs to evaluate associations between prenatal PFASs exposures and low birth weight and preterm birth. Maternal plasma concentrations were measured for six types of PFASs in early pregnancy. Several PFASs were associated with a reduction in birth weight and gestational age. We estimated a nearly 2-fold increase in risks of preterm birth for the higher quartiles of PFOA and perflourooctanesulfonate (PFOS) exposure. In spline models, risk of preterm birth was increased for perfluorononanoic acid (PFNA), perfluoroheptane sulfonate (PFHpS) and perfluorodecanoic acid (PFDA) in higher exposure ranges. We also observed some elevated risks for low birth weight but these estimates were less precise. Our findings strengthen the evidence that in-utero PFASs exposures affect fetal growth. Future studies are needed to evaluate whether these associations persist with the decline of PFOA and PFOS in populations and should also investigate newer types of fluorinated compounds introduced more recently.

## 1. Introduction

Perfluoroalkyl substances (PFASs) are industrial persistent pollutants that are widespread in the environment [1]. The most commonly used PFASs are perflourooctanesulfonate (PFOS), perfluorooctanote (PFOA), perfluorohexane sulfonate (PFHxS) and perfluorononanoic acid (PFNA) which have been detected nearly ubiquitously in human populations [1,2,3]. PFOS and PFOA have been phased out from production in the USA and Europe since the year 2000 because of concerns about possible harmful effects on human health. While the use of PFOS and PFOA are decreasing in some countries [3,4,5], they are still widely detectable. Also, human exposures to other type of PFASs such as PFNA have been reported to be increasing [3,4,5], and some newer types of fluorinated compounds, such as GenX (also named PFPrOPrA or HFPO–DA) [6], substitutes of PFOA, are now also detected in biota [6,7,8].

Animal studies have suggested that prenatal PFASs exposure can affect fetal growth i.e., PFOS and PFOA exposures in-utero can reduce birth weight and gestational age at delivery in rodents [9,10,11]. Several potential mechanisms have been suggested, including a disturbance of lipid and glucose homeostasis, effects on cell proliferation and differentiation, suppression of primary antibody responses, or altered glucocorticoids and reproductive hormones levels [12,13,14]. While high PFOS and PFOA exposures in pregnancy have been associated with lower average birth weights in human newborns in epidemiological studies [15,16,17,18], most studies analyzed small sample sizes and very few infants were born low birth weight or preterm. Moreover, there is only sparse evidence for the influence of other types of PFASs on fetal growth.

A previous study conducted in the Danish National Birth Cohort (DNBC) found an inverse association between maternal plasma PFOA levels and birth weight [15], however, this study only examined two types of PFASs (PFOS and PFOA), and even with a relatively large sample size of 1400 mother-child pairs there was insufficient power to evaluate adverse birth outcomes such as low birth weight and preterm birth. Here, we conducted additional analyses utilizing three DNBC sub-samples with a total of 3535 pregnancies, and aimed to reevaluate the previous findings. Additionally, we aimed to study associations between prenatal exposure to six types of PFASs, including PFOS, PFOA, PFHxS, PFNA, perfluoroheptane sulfonate (PFHpS) and perfluorodecanoic acid (PFDA) and adverse birth outcomes.

## 2. Materials and Methods

### 2.1. Study Population

The DNBC is a nationwide follow-up study of pregnant women and their offspring in Denmark [19]. Pregnant women were invited by their general practitioners from 1996 to 2002 and a total of 101,042 pregnancies were initially enrolled. About 50% of all pregnant women during the study period in Denmark were invited and 60% accepted. After informed consent, four computer-assisted telephone interviews based on structured questionnaires were conducted—approximately at the gestational weeks 12 and 30, and when the child was 6 and 18 months old. Two maternal blood samples were taken during pregnancy (once in the first and once in the second trimester), and one umbilical cord blood sample was obtained at birth and stored in a biobank. Blood samples were transported at room temperatures for about 4–48 h, but most samples arrived and were processed within 28 h.

**Figure 1:**
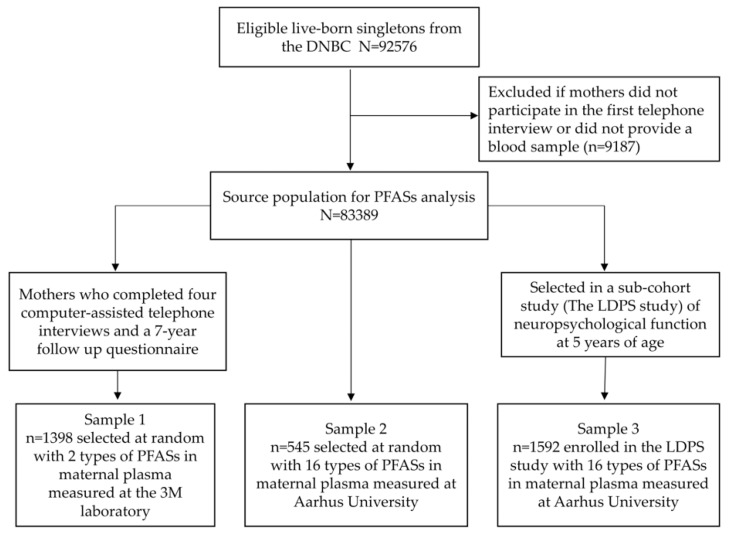
Selection of three DNBC sub-samples.

There were 92,576 live-born singletons in the DNBC after excluding unsuccessful pregnancies including abortions and stillbirths (*n* = 6207), non-singleton births (*n* = 2080), mother who emigrated (*n* = 51) or died (*n* = 3), unknown birth outcomes (*n* = 25), or missing dates of birth (*n* = 99), and one participant who withdrew from all pregnancies initially enrolled. The source population for this study is further limited to the 83,389 mother-child pairs who completed interview 1 and for whom maternal blood samples were available for PFASs analyses. Previously, three sub-studies have measured PFASs in maternal blood samples in the DNBC [15,20,21]. A selection flowchart for these samples is provided in Figure 1. Sample 1 included data for 1398 mothers-child pairs randomly selected among mothers who participated in all four telephone interviews and a 7-year follow up questionnaire [15]. Sample 2 included 545 control children selected at random from the DNBC cohort (after frequency matching to cases by sex; i.e., a boy to girl ratio of about 4:1) among those who completed interview 1 for a case-cohort study originally designed to study three neurodevelopmental disorders in children [20,22]. Sample 3 included 1592 participants enrolled in the Lifestyle During Pregnancy Study (LDPS) [21], a DNBC sub-cohort with a two-stage design and sampling strategies based on prenatal alcohol exposure categories with the overall aim to study early life influences of alcohol consumption on brain function in children at age 5 [21]. The research protocol for this study was approved by the Danish data inspectorate and the UCLA Institutional Review Board (IRB#16-001849).

### 2.2. Birh Outcomes

Birth weight (grams) and gestational age at birth were obtained from the National Hospital Discharge Register at the National Board of Health in Denmark. The information for gestational age recorded in the register was usually based on ultrasound examinations done before 24 weeks of gestation conducted by midwives (~97%) and only few were calculated from the first day of the last menstrual period (LMP). We excluded infants with extreme values of birth weight <500 g or >6800 g (*n* = 6) or gestational age <140 days or > 315 days (*n* = 4). Low birth weight (LBW) was defined as birth weight <2500 g [23], and preterm birth was defined as the birth of an infant before 37 completed weeks of gestation (259 days) following the World Health Organization definitions [24].

### 2.3. Exposure Assessment

Details about our analytic methods for PFASs have been described elsewhere [15,20,22]. Briefly, all blood samples collected in the DNBC were sent by mail to Statens Serum Institute in Copenhagen, separated and stored in freezers at −20 °C or −80 °C. For study sample 1, plasma concentrations of PFOS and PFOA were measured in the 3M Toxicology Laboratory [25], and samples in the study sample 2 and 3 were analyzed at the Department of Environmental Science at Aarhus University [22]. Samples taken out from the biobank were sorted in random order before sending them to the laboratories. A total of 0.1 mL stored maternal plasma was sent to the laboratories from the biobank. A Solid Phase Extraction (SPE) technique was used for sample extraction and purification. PFASs concentrations were measured by liquid chromatography–tandem mass spectrometry (LC-MS/MS). Only PFOS and PFOA were measured in sample 1 because they were the only compounds that could be measured in 2007. A total of 16 PFASs were measured in both samples 2 and 3 in 2011 and 2014, respectively. In samples 2 and 3, we focused on six types of PFASs that were found to be quantifiable in >90% of all measured samples including PFOS 100%, PFOA 100%, PFHxS 98%, PFHpS 96%, PFNA 92%, and PFDA 90% [20,22].

Comparisons of PFASs measurements in the two laboratories have previously been performed [22]. Although the absolute PFOS and PFOA values read-out from the 3M laboratory were found to be slightly higher than the Aarhus laboratory, the correlations of PFOS and PFOA concentrations measured in the same samples (*n* = 21) produced by the two laboratories were very high (Pearson correlation *r* = 0.94 for PFOS and *r* = 0.95 for PFOA).

### 2.4. Maternal and Newborn Covariates

Information on infant sex, infant birth year (as continuous variable), maternal age (19–29, 30–34, 35–39), parity (0, 1, >1) were collected from the medical register, while other potential confounders such as socio-occupational status (high, medium, low), pre-pregnancy body mass index (BMI; <18.5, 18.5–24.9, 25.0–29.9, ≥30), smoking during pregnancy (yes/no) and alcohol intake during pregnancy(never, ≤1/week, >1/week) was recorded in the highly structured questionnaires collected during pregnancy (available at http://www.bsmb.dk) [19]. Socio-occupational status was created based on self-reported maternal and paternal education and occupation using three categories (high, medium and low): higher education (four years beyond high school) or work in management were classified as high, skilled workers and middle-range education as medium, unskilled workers and unemployed as low status [26]. Although infant sex is unlikely to influence prenatal PFASs levels, adjusting for child’s sex might be important to account for live-birth selection bias since PFASs may affect fetal loss [27,28], and female and male could be disproportionally affected.

### 2.5. Statistical Analysis

We used multivariable linear regression to evaluate the expected differences in birth weight (grams) and length of gestation (days) according to maternal plasma PFASs level. In addition, we used multivariable logistic regression to estimate the adjusted odds ratio (OR) and 95% confidence interval (CI) for LBW and preterm birth according to PFASs exposures. The PFASs levels were analyzed as continuous values or categorized in quartiles. For continuous PFASs values, we analyzed log-transformed (base 2) PFASs exposure in the statistical models thus the exposure effect estimate represents an increase per doubling of the PFASs concentration (ng/mL). The PFASs quartile classifications were based on untransformed PFASs values and the lowest quartile was used as the reference. To further evaluate potential non-linear exposure-outcome responses, we fitted restricted cubic spline models with three knots at the 10th, 50th and 90th percentile of the PFASs value on LBW or preterm birth. We also allowed for higher flexibility (4 or 5 knots) in the spline models; but since results did not change substantially, we employed 3 knots to avoid over-fitting. To account for possible laboratory differences or “batch effects”, we added an indicator variable for study sample (1, 2 or 3) when we analyzed PFASs continuously, and we used the study-sample specific cut-off to generate PFASs quartiles.

All covariates except for infant birth year and gestational age at blood draw (weeks) were introduced into models as categorical variables (see Table 1 for the classifications for these variables). We used multiple imputations to account for the missing values for all above mentioned covariates (<10% of the sample had at least 1 missing value). PFASs values below the lower limit of quantification (LLOQ) were also replaced by multiple imputation algorithms (including the six PFASs and all above mentioned covariates in the model) when they were analyzed as continuous variables [20,22]. Values below LLOQ were classified in the lower quartile in categorical exposure analyses. Stratified analyses by parity, pre-pregnancy BMI and sex were performed to evaluate effect measure modification. Sex-specific associations between PFASs and fetal growth as well as modifying effects of PFASs on health by parity and maternal metabolic diseases have previously been suggested [17,29]. Tests for heterogeneity were performed by examining *p*-values for interaction term between each of the exposure and the potential modifier [30,31]. Moreover, we also conducted analyses separately for each study sample to examine the consistency of the results across strata. We employed weighted regression analysis throughout using the inverse-probability-weight (IPW) technique that accounted for the sampling fractions and also the participation probabilities from each of the study sub-samples. 

Details of the IPW have been described in previous studies [20,32,33]. The sampling probabilities were documented at the study design stage. Sample 2 was selected from the baseline source population, but sample 1 and 3 were selected conditioning on follow-up at age 7 (among invited ~60% participated) or in the LDPS sub-cohort (among invited ~50% participated) thus might be subject to bias due to non-participation during follow-up. The IPW included a range of factors measured for all women in the DNBC at baseline that were predictive of the participation status in follow-up, including maternal age, socio-occupational status, pre-pregnancy BMI, home size, planned pregnancy, and organic food intake during pregnancy. Birth outcomes such as preterm delivery and infants LBW were also included in the IPW model. Robust variance estimators were used to compute 95% CIs in all weighted regression analyses. A Pearson correlation matrix for the six PFASs is presented in the supplementary material (Table S1). To disentangle the possible effect for each of the PFASs, we constructed 3 multiple pollutants models considering the dimensionality of regression adjustment: model 1 co-adjusted for PFOS and PFOA (the most widespread PFASs that were measured in all samples), model 2 included those PFASs found to be correlated with birthweight or gestational age in single-pollutant models, and in model 3 we co-adjusted for all 6 types of PFASs.

In additional sensitivity analysis, we adjusted for fish intake (no, low, medium, high), and organic food consumption (never, rare, sometimes, often) during pregnancy to evaluate potential confounding by dietary factors [34]. We also utilized different cutoff points to define LBW (<2260 g or < 2650 g employing the 1st and the 3rd percentile of the birth weight distribution in the DNBC) and preterm birth (<35 or <36 completed gestational week). Because PFASs values measured in late pregnancy might be influenced by physiological factors such as changes in the blood volume or the glomerular filtration rate [35], we restricted the analyses using blood samples collected in the first pregnancy trimester only (92% of all samples). Finally, to adjust for gestational age when studying birth weight, we estimated the PFASs exposure effect on birth weight z-scores and on birth weight among term births only. Birth weight z-scores were calculated for boys and girls separately by their respective gestational week at birth (z-score = (observed birth weight value−mean)/standard deviation [SD]) [36]. All statistical analyses were performed using SAS version 9.4 (SAS Institute Inc., Cary, NC, USA) and STATA version 15 (StataCorp, College Station, TX, USA).

## 3. Results

Demographic and other characteristics of the study participants (unweighted) by study sample are presented in Table 1. As expected and due to over-sampling by design, there were more male infants in sample 2 and more women with alcohol intake during pregnancy in sample 3. The PFOS and PFOA values in sample 1 were slightly higher likely due to laboratory difference, while all PFASs levels in sample 2 and 3 were rather comparable. The distributions of birth weight and length of gestation were comparable in all samples, but the proportions of infants born LBW or preterm were slightly different across samples possibly due to differences in the sampling criteria.

In pooled analyses, we observed that per doubling of exposure in prenatal PFASs (ng/mL) specifically PFOS, PFOA, PFNA and PFHpS were associated with a 45 g, 36 g, 36 g or 39 g decrease in birth weight (Table 2). A greater reduction in birth weight was also observed with increasing PFASs quartiles for PFOA, PFNA and PFHpS, i.e., the estimated reduction in birth weight was more than 100 g for the highest quartile of PFOA and PFHpS compared with the lowest quartile. PFOS, PFOA, PFNA and PFHpS were also associated with a small decrease in gestational age in days at delivery, and similarly a larger effect size was observed when comparing the higher exposure quartiles to the lowest for each of the four compounds (Table 2). When we compare the effect estimate in each study sample, the point estimates for a doubling of PFASs exposures and birth weight were generally in the same direction, except for PFOA and PFHxS in sample 3 while the 95% CIs were wide (Table S2). However, the negative associations of PFASs on gestational age seem to be larger in sample 2 (Table S3). Overall, we did not detect strong modifying effects of maternal pre-pregnancy BMI, parity, and infant sex on the associations between prenatal PFASs and birth weight or gestational age (Tables S4 and S5). Nevertheless, some differences were observed such as a negative association between PFHxS and birth weight only in nulliparous women, and a negative association between PFNA and gestational age only in boys (*p*-value for interaction = 0.05). The negative associations of PFNA or PFDA on gestational age were also stronger among mothers with either lower or higher pre-pregnancy BMI compared with normal weight women (*p*-value for interaction ≤0.03).

Several positive associations between prenatal PFASs and preterm birth were observed (Table 3). The ORs for preterm birth were elevated in higher PFOS and PFOA quartiles i.e., an about 2-fold increase in the odds of preterm birth comparing the top three quartiles of PFOS and PFOA with the lowest one. 

**Figure 2:**
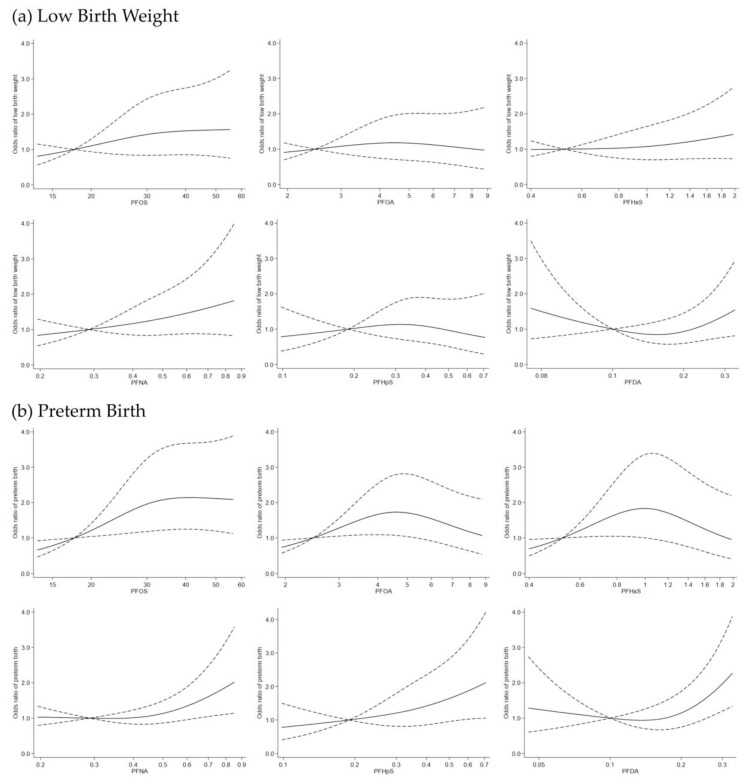
Odds ratio for low birth weight (**a**) and preterm birth (**b**) according to continuous PFASs values using a restricted cubic spline regression model with three knots at the 10th, 50th and 90th percentiles. The dashed lines represent 95% confidence intervals for the spline model (reference is the 10th percentile of each PFASs level). Model adjusted for a study sample indicator, infant sex, infant birth year, gestational week of blood draw, maternal age, parity, socio-occupational status, pre-pregnancy body mass index (BMI), smoking and alcohol intake during pregnancy. In graph (**a**), the *p*-values for non-linearity for PFOS, PFOA, PFHxS, PFNA, PFHpS and PFDA and low birth weight were 0.52, 0.43, 0.55, 0.88, 0.35, and 0.05, respectively. In graph (**b**), the *p*-values for non-linearity for PFOS, PFOA, PFHxS, PFNA, PFHpS and PFDA and preterm birth were 0.10, 0.01, 0.01, 0.05, 0.69, and 0.01, respectively.

Some non-linearity was also detected (*p*-value for non-linearity <0.10) for PFOA, PFHxS, PFNA, and PFDA in a spline model of exposure (Figure 2b). The estimated odds for preterm birth were increasing for PFOA and PFHxS from the lower to mid exposure ranges but then slightly decreased in the higher exposure range, while for PFNA, PFHpS and PFDA elevated odds ratio point estimates for preterm birth only appear in the higher exposure range. Some elevated ORs for LBW were also estimated in higher quartiles of PFOS and PFNA but for this outcome none of the estimates excluded the null value in the 95% CI (Table 3).

A non-linear exposure response for LBW was found for PFDA with a slight reduction of odds in the lower exposure range but estimated odds increasing again at higher exposure (>0.2 ng/mL) (Figure 2a). In sensitivity analyses, results remain unchanged in models that additionally adjust for dietary factors (Table S6). The association between PFASs and birth weight were attenuated when restricting to term births only, but the effect estimates (β) for PFOS, PFOA, PFNA and PFHpS were still in the negative direction ranging from −20.6 to −26.5 (Table S7). Moreover, the effect estimates (β) for birth weight z-scores were also negative for these four PFASs (Table S7). The correlations between most of the PFASs were moderate or high (*r* from 0.3 to 0.7) while PFOS and PFHpS were highly correlated (*r* = 0.89) (see Table S1 for the correlation matrix). We found that negative effect estimates of PFOS, PFNA and PFHpS on birth weight, and of PFNA or PFHpS on gestational age persisted upon co-pollutant adjustment (Table S8), even though the confidence intervals in multiple pollutants models were wide. Our findings did not change considerably when we chose alternative cut-off points to define LBW and preterm birth (Table S9).

The positive effect estimates between several PFASs and LBW were slightly strengthened using <2650 g as the cut-off while the results became very imprecise with the <2260 g cut-off due to the small number of cases. For preterm birth, the results were also slightly strengthened for PFNA and PFDA using <36 weeks as the cut-off but the effect estimates also became less precise using a <35 weeks cut-off. Our results also remained similar when restricting to samples collected in the first trimester only (data not shown).

## 4. Discussion

In this large combined sample from a prospective cohort study in Denmark, prenatal PFASs were generally inversely associated with birth weight and gestational age. Moreover, we found that prenatal exposures to several PFASs may increase the risks for preterm birth, and possibly also LBW but the estimates for LBW were imprecise. Spline models for exposure suggested possible effect on adverse birth outcomes at higher exposure ranges for the less prevalent PFASs in our samples such as PFNA, PFHpS and PFDA. This might be of concerns if these exposures rise in populations with changes in consumer product use of these chemicals [3,4,5].

An earlier study in the DNBC [15] was among the first to evaluate the associations between prenatal PFASs exposure and fetal growth indicators. At that time, only PFOS and PFOA were measurable in the laboratory, and the study reported that only prenatal PFOA, but not PFOS, was inversely associated with a small reduction in birth weight (adjusted β = −10.6, 95% CI −20.8 to −0.5 g for each ng/mL increase in PFOA). Although the estimated risks were found to be elevated for preterm birth or LBW in the study (ORs ranged from 1.4 to 6.0 for PFOS and PFOA quartile based analyses), CIs of the risk estimates were very wide due to the small number of cases (24 LBW, 53 preterm) included. Thus, we conducted this pooled analysis that utilizes additional PFASs samples from later measurements now available in the DNBC and re-evaluated earlier findings for fetal growth and birth outcomes. The results for study sample 1 (same data analyzed in Fei et al. [15] alone is generally consistent with the previous report. However, the effect sizes are not directly comparable since we estimated effect per log_2_ ng/mL exposure and additionally accounted for possible selection bias due to the sample selection criteria employed.

Other cohort studies that investigated the associations regarding prenatal PFASs exposures on fetal growth indicators were mostly small in size and they only assessed PFOS or PFOA exposures [37,38]. Among the larger cohorts (i.e., >500 births), two also reported that prenatal PFOA and PFNA exposures were associated with lower average birth weights [16,17], while several others reported non-statistically significant associations [29,39,40]. LBW and preterm birth were less studied possible due to insufficient sample size. The INMA cohort in Spain included 1202 mother-child pairs to study four types of PFASs (PFOS, PFOA, PFHxS and PFNA) and reported that high PFOS exposure was associated with LBW (OR 1.90, 95% CI 0.98, 3.68) in boys (618, 25 LBW cases) [29]. The Project VIVA cohort of 1645 participants with 120 preterm birth cases in Eastern Massachusetts (USA) studied the same four PFASs and reported that PFOS and PFNA were associated with higher odds of preterm birth (adjusted OR 2.4, 95% CI 1.3, 4.4 comparing the highest PFOS quartile with the lowest) [17]. Differences in PFASs concentrations and mixtures, timing of sample collection (ranging from early pregnancy to cord blood), statistical models, and sample size could have contributed to inconsistency in results and should be considered when comparing findings across studies [37].

Physiologically, animal studies have shown the negative effect of prenatal PFASs exposures on birth outcomes and several potential mechanisms have been suggested [11,41,42]. PFASs cross the placenta [43], and they may impair fetal growth and development through activating the peroxisome proliferator-activated receptor alpha which regulates lipid and glucose homeostasis [44]. In addition, PFASs were reported to interfere with thyroid function and reproductive hormones biosynthesis, and these hormones might be critical for fetal development during pregnancy [45,46]. Additional molecular and epidemiologic investigations are still needed to evaluate the contribution of these mechanisms for each of the PFASs on fetal growth and birth outcomes.

Our study has several strengths. All three sub-samples were selected from a nationwide well-described cohort of pregnant women and their infants [19]. The PFASs measures were obtained using state-of-the-art laboratory facilities, and the laboratory personnel were blinded to exposure and outcome status. Data on birth weight and gestational age originated from the Danish Hospital Discharge Register that relied on standard clinic procedures. More importantly, we took full advantage of the existing PFASs measures generated in the DNBC and conducted this pooled analysis with a sample size sufficient to evaluate some adverse birth outcomes that were not well studied previously. Statistical power increased considerably with data pooling. For instance, PFOS was negatively related to birth weight in each sub-sample, but only in pooled analyses that included all three samples the effect estimate reached the conventional statistical significance level (Table S2). Enhanced statistical power helps to stabilize effect estimates and allows us to detect smaller size effects for such ubiquitous environmental contaminants that affect large populations. 

The effect estimates were largely consistent across study samples, but some variations were observed. This could be due to different sampling and selection criteria employed to generate each study sub-sample, or the influence of measurement errors or simply of chance. We adjusted for sampling and selection probabilities using weighted regressions throughout, but some differences in study characteristics across samples may still have remained. A moderate to high correlation between different PFASs make it difficult to disentangle specific exposure effects for each chemical from the effect of the mixtures. Our large sample size allowed us to conduct multi-pollutants analyses, and generally the estimated exposure effects for several PFASs compounds persisted upon co-pollutant adjustments. Some advanced statistical methods for mixture analyses have been proposed [47,48], but these methods have limitations such as they might be better suited as prediction models to screen for a wide range of chemicals from different sources, and the interpretation of results might become less straight forward due to the necessary standardization of exposure values. Moreover, bias amplification can occur in co-pollutants or mixture analyses in the presence of uncontrolled confounding [49]. Future research is needed to explore mixture effects possibly coupled with bias analyses that also consider biological interactions of the PFASs compounds that need to be derived from experimental models [46].

The observed association may not be causal and can be influenced by bias. There could be unmeasured factors we could not take into considerations leading to residual confounding. When using biomarkers of PFASs, physiological factors that affect accumulation or excretions of PFASs should also be considered. For instance, lower glomerular filtration rate (GFR) in mid- or late-pregnancy has been suggested to be such a possible confounding factor [35]. Mothers with lower GFR might possibly have lower PFASs excretion and higher PFASs plasma levels, and a lower GFR in pregnancy has been linked with adverse birth outcomes. However, our PFASs measures are taken in first trimester plasma samples and PFASs measures in early pregnancy are less likely to be influenced by changes of GFR in pregnancy [35]. Recent studies have also shown that adjusting for GFR and plasma albumin did not materially change the associations between PFASs measured in early pregnancy and birth outcomes [17,29]. We did not have data for other persistent and non-persistent organic pollutants such as polychlorinated biphenyls or phthalates, thus we could not evaluate potential confounding by these chemicals that may also influence fetal growth. However, in other populations the correlations between PFASs and these other chemicals were not very high [50,51], which is expected because the exposure sources might be quite different.

Any measurement errors in exposures and outcomes are expected to be non-differential and might bias the associations mostly towards the null. Participants were unlikely to be aware of their PFASs levels which limit the possibility of self-selection bias. However, prenatal PFASs exposures may increase risk of miscarriages [27,52]. Our previous study has demonstrated that “live-birth selection bias” may occur if PFASs cause fetal loss and only infants born alive are studied. In certain scenarios, true effect estimates might be under-estimated but a spurious association could also be created even when PFASs has no true causal effect on the outcomes [28].

## 5. Conclusions

In conclusion, our analysis using three sub-samples from the DNBC demonstrated that several prenatal PFASs are inversely associated with birth weight and gestational age, and prenatal exposure to several PFASs may increase the risks for preterm birth. Our findings strengthen the evidence that in-utero PFASs exposures might affect fetal growth. Exposure levels of PFOS and PFOA are declining in some western countries but they are still widely detected. Adverse effects on birth outcomes in our samples were also observed in the higher exposure ranges of some of the less commonly detected PFASs such as PFNA and PFDA which might be concerning if exposures to these compounds increase in populations. Considering the ubiquity of PFASs contamination in the environment and in humans, strategies and efforts to prevent PFASs exposures in pregnant women and young infants should continue. Further study is also needed to evaluate whether these associations persist with lower exposure levels for PFOS and PFOA, and substitutes and newer types of fluorinated compounds need to be scrutinized as well.

## Figures and Tables

**Table 1:** Maternal and newborn characteristics of sub-study participants (*n* = 3535) from the DNBC (1996–2002).

	Median (Interquartile Range) or *N* (%)			
	Total	Sample 1	Sample 2	Sample 3
**Maternal Characteristics**				
PFAS (ng/mL)				
PFOS	30.1 (22.9–39.0)	33.4 (26.1–43.3)	27.4 (20.4–35.6)	28.1 (21.6–35.8)
PFOA	4.6 (3.3–6.0)	5.2 (3.9–7.0)	4.0 (3.0–5.4)	4.3 (3.2–5.5)
PFHxS	1.0 (0.7–1.3)	N/A	0.9 (0.7–1.2)	1.1 (0.8–1.4)
PFNA	0.5 (0.4–0.6)	N/A	0.4 (0.4–0.6)	0.5 (0.4–0.6)
PFHpS	0.4 (0.3–0.5)	N/A	0.3 (0.2–0.4)	0.4 (0.3–0.5)
PFDA	0.2 (0.1–0.2)	N/A	0.2 (0.1–0.2)	0.2 (0.1–0.2)
Age (years)				
19–29	1638 (46.3)	664 (47.5)	273 (50.1)	701 (44.0)
30–34	1321 (37.4)	504 (36.1)	200 (36.7)	617 (38.8)
35–39	576 (16.3)	230 (16.4)	72 (13.2)	274 (17.2)
Socio-occupational status				
High	2366 (67.2)	899 (64.5)	336 (61.9)	1131 (71.3)
Medium	1057 (30.0)	453 (32.5)	192 (35.4)	412 (26.0)
Low	100 (2.8)	42 (3.0)	15 (2.7)	43 (2.7)
Missing	12	4	2	6
Parity				
0	1622 (47.1)	607 (44.4)	245 (46.2)	770 (49.8)
1	1212 (35.2)	498 (36.4)	209 (39.4)	505 (32.7)
>1	610 (17.7)	263 (19.2)	76 (14.4)	271 (17.5)
Missing	91	30	15	46
Alcohol intake during pregnancy				
Never	766 (21.7)	400 (28.6)	159 (29.2)	207 (13.0)
≤1 per week	629 (17.8)	352 (25.2)	139 (25.5)	138 (8.7)
>1 per week	2140 (60.5)	646 (46.2)	247 (45.3)	1247 (78.3)
Smoking during pregnancy				
No	2534 (71.7)	1050 (75.1)	407 (74.7)	1077 (67.7)
Yes	1001 (28.3)	348 (24.9)	138 (25.3)	515 (32.3)
Pre-pregnancy BMI (kg/m^2^)				
<18.5	143 (4.1)	58 (4.3)	24 (4.5)	61 (3.9)
18.5–24.9	2355 (68.0)	904 (66.4)	358 (66.4)	1093 (70.0)
25.0–29.9	705 (20.4)	298 (21.9)	115 (21.3)	292 (18.7)
≥30.0	258 (7.5)	101 (7.4)	42 (7.8)	115 (7.4)
Missing	74	37	6	31
**Newborn Characteristics**				
Sex				
Female	1559 (44.1)	688 (49.2)	110 (20.2)	761 (47.8)
Male	1976 (55.9)	710 (50.8)	435 (79.8)	831 (52.2)
Weight (g)	3600 (3270–3960)	3630 (3260–4000)	3628 (3250–3970)	3600 (3280–3925)
Gestational age (days)	281 (275–288)	281 (274–288)	281 (274–287)	282 (275–288)
Low birth weight				
Yes	61 (1.7)	24 (1.7)	14 (2.6)	23 (1.5)
No	3446 (98.3)	1363 (98.3)	526 (97.4)	1557 (98.5)
Missing	28	11	5	12
Preterm birth				
Yes	112 (3.2)	53 (3.8)	17 (3.1)	42 (2.6)
No	3410 (96.8)	1337 (96.2)	528 (96.9)	1545 (97.4)
Missing	13	8	0	5

**Table 2:** Adjusted differences (β) and 95% confidence intervals (CI) for birth weight in grams and gestational age in days according to prenatal PFASs exposure levels.

Exposure Level ^b^	Birth Weight		Gestational Age	
	*n*	Adjusted Difference ^a^ in Birth Weight (β and 95%CI)	*n*	Adjusted Difference ^a^ in Gestational Age (β and 95%CI)
Pooled sample 1, 2 and 3				
PFOS				
Per doubling of exposure	3507	−45.2 (−76.8, −13.6)	3522	−1.1 (−1.7, −0.4)
Q1	885	ref	889	ref
Q2	875	24.7 (−24.8, 74.1)	879	−1.1 (−2.1, −0.1)
Q3	872	−50.1 (−101.1, 0.9)	877	−2.0 (−3.1, −1.0)
Q4	875	−48.2 (−99.0, 2.5)	877	−1.5 (−2.6, −0.5)
PFOA				
Per doubling of exposure	3507	−35.6 (−66.3, −5.0)	3522	−0.4 (−1.0, 0.3)
Q1	885	ref	888	ref
Q2	873	−20.4 (−70.0, 29.2)	874	−1.4 (−2.4, −0.3)
Q3	873	−25.9 (−77.7, 25.9)	878	−1.2 (−2.2, −0.1)
Q4	876	−117.0 (−172.3, −61.6)	882	−1.7 (−2.9, −0.6)
Pooled sample 2 and 3				
PFHxS				
Per doubling of exposure	2120	1.2 (−28.3, 30.7)	2132	−0.2 (−0.8, 0.4)
Q1	535	ref	537	ref
Q2	544	37.3 (−25.7, 100.2)	545	−1.2 (−2.5, 0.1)
Q3	510	7.6 (−58.1, 73.2)	519	−0.4 (−1.7, 1.0)
Q4	531	8.6 (−59.7, 76.9)	531	−0.9 (−2.3, 0.5)
PFNA				
Per doubling of exposure	2120	−36.3 (−70.6, −2.0)	2132	−1.0 (−1.7, −0.3)
Q1	556	ref	562	ref
Q2	537	−9.1 (−71.6, 53.3)	536	−1.4 (−2.7, −0.2)
Q3	513	−21.7 (−86.3, 42.8)	519	−1.1 (−2.4, 0.2)
Q4	514	−81.2 (−147.1, −15.4)	515	−1.5 (−2.8, −0.2)
PFHpS				
Per doubling of exposure	2120	−38.9 (−72.6, −5.1)	2132	−1.2 (−1.9, −0.5)
Q1	547	ref	552	ref
Q2	520	−62.1 (−124.6, 0.4)	521	−1.7 (−3.0, −0.4)
Q3	538	−110.8 (−177.7, −43.8)	542	−2.6 (−4.0, −1.3)
Q4	515	−102.6 (−169.0, −36.2)	517	−2.0 (−3.3, −0.7)
PFDA				
Per doubling of exposure	2120	−9.0 (−43.2, 25.2)	2132	−0.6 (−1.3, 0.1)
Q1	655	ref	660	ref
Q2	456	−22.6 (−87.2, 42.1)	462	0.6 (−0.7, 1.9)
Q3	518	16.3 (−45.3, 77.9)	517	−0.2 (−1.4, 1.1)
Q4	491	−16.0 (−80.0, 47.9)	493	−0.5 (−1.8, 0.8)

^a^: Adjusted for infant sex, infant birth year, gestational week of blood draw, maternal age, parity, socio-occupational status, pre-pregnancy body mass index (BMI), smoking and alcohol intake during pregnancy. ^b^: For continuous PFASs a study sample indicator was included in the regression model and for the estimation of PFASs quartile effects the sample-specific quartile cut-off was utilized.

**Table 3:** Adjusted Odds Ratios (OR) and 95% confidence intervals (CI) for low birth weight and preterm birth according to prenatal PFASs exposure levels.

Exposure Level ^b^	Low Birth Weight		Preterm Birth	
	*n*	Adjusted OR ^a^ and 95% CI	*n*	Adjusted OR ^a^ and 95% CI
Pooled sample 1,2 and 3				
PFOS				
Per doubling of exposure	61	1.3 (0.9, 2.0)	112	1.5 (1.1, 2.2)
Q1	10 (1.1%)	ref	19 (2.1%)	ref
Q2	16 (1.8%)	1.4 (0.7, 2.8)	28 (3.2%)	2.0 (1.1, 3.6)
Q3	16 (1.8%)	1.8 (0.9, 3.6)	37 (4.2%)	3.3 (1.8, 5.8)
Q4	19 (2.2%)	1.2 (0.6, 2.4)	28 (3.2%)	1.9 (1.0, 3.5)
PFOA				
Per doubling of exposure	61	1.0 (0.7, 1.5)	112	1.1 (0.8, 1.5)
Q1	12 (1.4%)	ref	18 (2.0%)	ref
Q2	14 (1.6%)	1.5 (0.8, 3.1)	32 (3.7%)	3.2 (1.8, 5.6)
Q3	13 (1.5%)	1.2 (0.5, 2.5)	31 (3.5%)	1.7 (0.9, 3.2)
Q4	22 (2.5%)	1.5 (0.7, 3.3)	31 (3.5%)	1.9 (1.0, 3.6)
Pooled sample 2 and 3				
PFHxS				
Per doubling of exposure	37	1.2 (0.8, 1.7)	59	1.1 (0.8, 1.5)
Q1	9 (1.7%)	ref	13 (2.4%)	ref
Q2	11 (2.0%)	1.1 (0.5, 2.4)	18 (3.3%)	2.3 (1.1, 4.6)
Q3	5 (1.0%)	0.5 (0.2, 1.3)	14 (2.7%)	1.5 (0.7, 3.2)
Q4	12 (2.3%)	0.8 (0.4, 1.9)	14 (2.6%)	1.0 (0.5, 2.3)
PFNA				
Per doubling of exposure	37	1.5 (0.9, 2.4)	59	1.4 (0.9, 2.1)
Q1	9 (1.6%)	ref	13 (2.3%)	ref
Q2	4 (0.7%)	1.0 (0.4, 2.5)	14 (2.6%)	1.2 (0.6, 2.5)
Q3	11 (2.1%)	1.4 (0.6, 3.2)	14 (2.7%)	0.9 (0.4, 1.9)
Q4	13 (2.5%)	1.5 (0.6, 3.6)	18 (3.5%)	1.7 (0.8, 3.3)
PFHpS				
Per doubling of exposure	37	1.0 (0.6, 1.5)	59	1.5 (1.0, 2.1)
Q1	12 (2.2%)	ref	16 (2.9%)	ref
Q2	7 (1.3%)	1.1 (0.5, 2.4)	8 (1.5%)	1.5 (0.7, 3.0)
Q3	8 (1.5%)	1.2 (0.5, 2.6)	19 (3.5%)	1.6 (0.7, 3.3)
Q4	10 (1.9%)	0.5 (0.2, 1.4)	16 (3.1%)	1.8 (0.8, 3.7)
PFDA				
Per doubling of exposure	37	1.2 (0.8, 1.9)	59	1.7 (1.2, 2.5)
Q1	13 (2.0%)	ref	18 (2.7%)	ref
Q2	7 (1.5%)	0.8 (0.4, 1.8)	9 (1.9%)	1.0 (0.5, 2.0)
Q3	4 (0.8%)	0.4 (0.2, 1.0)	15 (2.9%)	1.1 (0.5, 2.1)
Q4	13 (2.6%)	0.9 (0.4, 2.0)	17 (3.4%)	1.6 (0.8, 3.0)

^a^: Adjusted for infant sex, infant birth year, gestational week of blood draw, maternal age, parity, socio-occupational status, pre-pregnancy body mass index (BMI), smoking and alcohol intake during pregnancy. ^b^: For continuous PFASs a study sample indicator was included in the regression model and for the estimation of PFASs effects by quartile the sample-specific quartile cut-off was utilized.

## Supplementary Materials

The following are available online at http://www.mdpi.com/1660-4601/15/9/1832/s1, Table S1: Pearson Correlation Coefficients between the PFASs (ng/mL), Table S2: Adjusted differences (β) and 95% confidence intervals (CI) for birth weight in grams per doubling of prenatal PFASs levels, stratified by study sample, Table S3: Adjusted differences (β) and 95% confidence intervals (CI) for gestational age in days per doubling of prenatal PFASs levels, stratified by study sample, Table S4: Adjusted differences (β) and 95% confidence intervals (CI) for birth weight in grams per doubling of prenatal PFASs levels, stratified by potential effect modifiers, Table S5: Adjusted differences (β) and 95% confidence intervals (CI) for gestational age in days per doubling of prenatal PFASs levels, stratified by potential effect modifiers, Table S6: Differences (β) and 95% confidence intervals (CI) for birth weight per doubling of prenatal PFASs levels additionally adjusted for dietary factors, Table S7: Adjusted differences (β) and 95% confidence intervals (CI) for birth weight in grams among all or term birth and birth weight Z-score per doubling of prenatal PFASs levels, Table S8: Adjusted differences (β) and 95% confidence intervals (CI) for birth weight in grams and gestational age in days per doubling of prenatal PFASs levels mutually adjusting for different PFASs, Table S9: Adjusted Odds Ratios (OR) and 95% confidence intervals(CI) for low birth weight and preterm birth according to different cutoff points and per doubling of prenatal PFASs levels.
