# Supplementary material for: Prenatal Exposure to Perfluoroalkyl Substances and Birth Outcomes; An Updated Analysis from the Danish National Birth Cohort
Source: Int J Environ Res Public Health. 2018 Aug 24;15(9):1832. doi: 10.3390/ijerph15091832 (PMC6164159; doi:10.3390/ijerph15091832)
Supplement: Supplementary file 1 [file ijerph-15-01832-s001.pdf]

Supplementary Information

Prenatal Exposure to Perfluoroalkyl Substances and Birth Outcomes; an Updated Analysis from the Danish National Birth Cohort

Table S1. Pearson Correlation Coefficients between the PFASs (ng/mL) [a].

	PFOS	PFOA	PFHxS	PFNA	PFHpS
**PFOA**	0.66				
**PFHxS**	0.30	0.33			
**PFNA**	0.48	0.47	0.28		
**PFHpS**	0.89	0.67	0.39	0.54	
**PFDA**	0.48	0.28	0.17	0.73	0.49

[a] Based on 3,535 samples for PFOS and PFOA, and 2,137 samples for PFHxS, PFNA, PFHpS and PFDA.

Table S2. Adjusted differences (β) and 95% confidence intervals (CI) for birth weight in grams per doubling of prenatal PFASs levels, stratified by study sample.

No. of Subjects	Adjusted Difference in Birth Weight (β and 95%CI) [a]			
	**Pooled** [b]	**Sample1**	**Sample2**	**Sample3**
	3,507	1,387	540	1,580
**PFOS**	-45.2 (-76.8, -13.6)	-30.0 (-81.9, 21.9)	-51.0 (-127.6, 25.7)	-40.5 (-88.0, 7.0)
**PFOA**	-35.6 (-66.3, -5.0)	-54.1 (-102.7, -5.5)	-68.7 (-146.5, 9.0)	7.0 (-39.5, 53.6)
**PFHxS**	1.2 (-28.3, 30.7)	N/A	-49.1 (-99.6, 1.5)	31.7 (-4.7, 68.1)
**PFNA**	-36.3 (-70.6, -2.0)	N/A	-9.7 (-75.2, 55.7)	-41.7 (-82.5, -1.0)
**PFHpS**	-38.9 (-72.6, -5.1)	N/A	-52.5 (-112.5, 7.4)	-26.1 (-67.4, 15.3)
**PFDA**	-9.0 (-43.2, 25.2)	N/A	-8.9 (-66.7, 48.8)	-5.3 (-48.1, 37.6)

[a] Adjusted for infant sex, infant birth year, gestational week of blood draw, maternal age, parity, socio-occupational status, pre-pregnancy body mass index (BMI), smoking and alcohol intake during pregnancy. [b] We additionally adjusted for a study sample indicator in the model.

Table S3. Adjusted differences (β) and 95% confidence intervals (CI) for gestational age in days per doubling of prenatal PFASs levels, stratified by study sample.

	Adjusted difference in gestational age (β and 95%CI) [a]			
	**Pooled** [b]	**Sample1**	**Sample2**	**Sample3**
**No. of subjects**	3,522	1,390	545	1,587
**PFOS**	-1.1 (-1.7, -0.4)	-0.6 (-1.7, 0.5)	-3.2 (-4.8, -1.6)	-0.5 (-1.5, 0.4)
**PFOA**	-0.4 (-1.0, 0.3)	-0.1 (-0.2, 0.9)	-2.3 (-3.9, -0.7)	0.4 (-0.6, 1.3)
**PFHxS**	-0.2 (-0.8, 0.4)	N/A	-1.3 (-2.4, -0.3)	0.5 (-0.3, 1.2)
**PFNA**	-1.0 (-1.7, -0.3)	N/A	-0.9 (-2.2, 0.5)	-1.1 (-1.9, -0.2)
**PFHpS**	-1.2 (-1.9, -0.5)	N/A	-2.5 (-3.7, -1.2)	-0.6 (-1.5, 0.2)
**PFDA**	-0.6 (-1.3, 0.1)	N/A	-1.3 (-2.5, -0.1)	-0.3 (-1.2, 0.5)

[a] Adjusted for infant sex, infant birth year, gestational week of blood draw, maternal age, parity, socio-occupational status, pre-pregnancy body mass index (BMI), smoking and alcohol intake during pregnancy. [b] We additionally adjusted for a study sample indicator in the model.

Table S4. Adjusted differences (β) and 95% confidence intervals (CI) for birth weight in grams per doubling of prenatal PFASs levels, stratified by potential effect modifiers.

Adjusted differences in birth weight (β and 95%CI) [a], pooled samples 1, 2 and 3				Adjusted differences in birth weight (β and 95%CI) [a], pooled samples 2 and 3				
**Strata**	**No.**	**PFOS**	**PFOA**	**No.**	**PFHxS**	**PFNA**	**PFHpS**	**PFDA**
**All**	3,507	-45.2 (-76.8, -13.6)	-35.6 (-66.3, -5.0)	2,120	1.2 (-28.3, 30.7)	-36.3 (-70.6, -2.0)	-38.9 (-72.6, -5.1)	-9.0 (-43.2, 25.2)
**Sex**								
Female	1,547	-65.3 (-111.7, -18.9)	-25.0 (-71.4, 21.5)	865	-4.4 (-48.3, 39.5)	-26.5 (-78.9, 26.0)	-61.0 (-112.9, -9.2)	-16.5 (-66.3, 33.3)
Male	1,960	-24.3 (-67.1, 18.6)	-41.5 (-82.1, -0.9)	1,255	14.0 (-25.6, 53.7)	-44.6 (-90.2, 1.0)	-19.4 (-63.6, 24.9)	-2.3 (-49.0, 44.5)
p for interaction		0.31	0.39		0.92	0.29	0.52	0.79
**Parity**								
Nulliparous	1,653	-38.5 (-84.0, 7.0)	-29.7 (-77.4, 18.0)	1,039	-47.0 (-86.4, -7.5)	-37.8 (-94.0, 18.4)	-58.1 (-105.8, -10.3)	-39.8 (-87.2, 7.6)
Parous	1,854	-57.8 (-101.7, -14.0)	-58.6 (-98.9, -18.3)	1,081	10.2 (-34.1, 54.5)	-49.0 (-93.3, -4.8)	-46.1 (-93.2, 1.0)	20.1 (-29.4, 69.6)
p for interaction		0.89	0.75		0.05	0.98	0.18	0.14
**BMI**								
<18.5	150	58.2 (-73.5, 189.8)	-12.7 (-161.9, 136.4)	88	-11.1 (-147.3, 125.2)	-104.5 (-240.2, 31.1)	-7.0 (-171.7, 157.8)	-78.3 (-244.0, 87.3)
p for interaction [b]		0.11	0.61		0.93	0.63	0.88	0.19
18.5-24.9	2,379	-53.5 (-90.0, -17.0)	-40.9 (-75.8, -6.0)	1,459	-6.1 (-41.4, 29.3)	-32.7 (-74.6, 9.2)	-50.1 (-89.7, -10.5)	11.9 (-28.6, 52.4)
≥25.0	978	-53.4 (-121.3, 14.5)	-23.0 (-89.5, 43.4)	573	3.4 (-56.4, 63.2)	-29.9 (-104.6, 44.7)	-19.3 (-89.2, 50.5)	-32.6 (-103.4, 38.2)
p for interaction [b]		0.43	0.41		0.43	0.60	0.74	0.21

[a] Adjusted for a study sample indicator, infant sex, infant birth year, gestational week of blood draw, maternal age, parity, socio-occupational status, pre-pregnancy body mass index (BMI), smoking and alcohol intake during pregnancy. b P-values for interaction were calculated in reference to the 'normal' BMI (18.5-24.9) group.

Table S5. Adjusted differences (β) and 95% confidence intervals (CI) for gestational age in days per doubling of prenatal PFASs levels, stratified by potential effect modifiers.

	Adjusted difference in gestational age (β and 95%CI) [a], pooled sample 1, 2 and 3			Adjusted difference in gestational age (β and 95%CI) [a], pooled sample 2 and 3				
**Strata**	**No.**	**PFOS**	**PFOA**	**No.**	**PFHxS**	**PFNA**	**PFHpS**	**PFDA**
**All**	3,522	-1.1 (-1.7, -0.4)	-0.4 (-1.0, 0.3)	2,132	-0.2 (-0.8, 0.4)	-1.0 (-1.7, -0.3)	-1.2 (-1.9, -0.5)	-0.6 (-1.3, 0.1)
**Sex**								
Female	1,553	-1.0 (-2.0, 0.0)	-0.1 (-1.1, 0.9)	869	0.1 (-0.8, 1.0)	0.0 (-1.0, 1.1)	-1.1 (-2.1, 0.0)	0.1 (-0.9, 1.1)
Male	1,969	-1.1 (-2.0, -0.3)	-0.6 (-1.4, 0.3)	1,263	-0.4 (-1.2, 0.4)	-1.9 (-2.8, -1.0)	-1.3 (-2.2, -0.4)	-1.2 (-2.1, -0.2)
p for interaction		0.72	0.83		0.50	0.05	1.00	0.18
**Parity**								
0	1,660	-1.2 (-2.2, -0.2)	-0.2 (-1.2, 0.9)	1,045	-0.6 (-1.5, 0.2)	-0.4 (-1.6, 0.8)	-1.3 (-2.3, -0.3)	-0.5 (-1.5, 0.5)
≥1	1,862	-1.0 (-1.8, -0.1)	-0.5 (-1.3, 0.2)	1,087	-0.2 (-1.1, 0.6)	-1.3 (-2.2, -0.5)	-1.3 (-2.2, -0.4)	-0.5 (-1.5, 0.4)
p for interaction		0.22	0.90		0.48	0.38	0.37	0.95
**BMI**								
<18.5	150	-0.5 (-3.8, 2.8)	-2.2 (-5.9, 1.5)	88	-0.9 (-4.9, 3.1)	-4.8 (-8.7, -0.9)	-0.9 (-5.7, 3.8)	-3.2 (-8.0, 1.6)
p for interaction [b]		0.79	0.13		0.94	0.03	0.86	0.01
18.5-24.9	2,393	-0.7 (-1.5, 0.0)	0.0 (-0.7, 0.7)	1,469	-0.4 (-1.1, 0.3)	0.0 (-0.9, 0.8)	-1.1 (-1.9, -0.3)	0.5 (-0.4, 1.3)
≥25.0	979	-1.8 (-3.2, -0.5)	-0.3 (-1.7, 1.0)	575	0.5 (-0.6, 1.5)	-1.5 (-2.9, -0.2)	-1.0 (-2.3, 0.2)	-1.7 (-3.0, -0.5)
p for interaction [b]		0.05	0.25		0.43	0.01	0.80	0.00

[a] Adjusted for a study sample indicator, infant sex, infant birth year, gestational week of blood draw, maternal age, parity, socio-occupational status, pre-pregnancy body mass index (BMI), smoking and alcohol intake during pregnancy. [b] P-values for interaction were calculated in reference to the 'normal' BMI (18.5-24.9) group.

Table S6. Differences (β) and 95% confidence intervals (CI) for birth weight per doubling of prenatal PFASs levels additionally adjusted for dietary factors.

		Low birth weight		Preterm birth	
		Model 1[a]	Model 2[b]	Model 1[a]	Model 2[b]
Adjusted OR and 95%CI, pooled sample 1, sample 2 and 3	**No. Cases**	61	61	112	112
	**PFOS**	1.3 (0.9, 2.0)	1.3 (0.8, 1.9)	1.5 (1.1, 2.2)	1.6 (1.1, 2.2)
	**PFOA**	1.0 (0.7, 1.5)	1.0 (0.7, 1.5)	1.1 (0.8, 1.5)	1.2 (0.8, 1.6)
Adjusted OR and 95%CI, pooled sample 2 and 3	**No. Cases**	37	37	59	59
	**PFHxS**	1.2 (0.8, 1.7)	1.2 (0.8, 1.8)	1.1 (0.8, 1.5)	1.1 (0.8, 1.5)
	**PFNA**	1.5 (0.9, 2.4)	1.6 (0.9, 2.6)	1.4 (0.9, 2.1)	1.5 (1.0, 2.2)
	**PFHpS**	1.0 (0.6, 1.5)	1.0 (0.6, 1.5)	1.5 (1.0, 2.1)	1.5 (1.1, 2.2)
	**PFDA**	1.2 (0.8, 1.9)	1.3 (0.8, 2.1)	1.7 (1.2, 2.5)	1.8 (1.2, 2.6)

[a] Adjusted for a study sample indicator, infant sex, infant birth year, gestational week of blood draw, maternal age, parity, socio-occupational status, pre-pregnancy body mass index (BMI), smoking and alcohol intake during pregnancy. [b] Adjusted for a study sample indicator, infant sex, infant birth year, gestational week of blood draw, maternal age, parity, socio-occupational status, pre-pregnancy body mass index (BMI), smoking, alcohol intake during pregnancy, fish intake and ecological/organic food consumption during pregnancy.

Table S7. Adjusted differences (β) and 95% confidence intervals (CI) for birth weight in grams among all or term birth and birth weight Z-score per doubling of prenatal PFASs levels.

		**Adjusted differences in birth weight (β and 95%CI)**		**Adjusted differences in birth weight among term birth only (β and 95%CI)**		**Adjusted differences in Z-score[c] (β and 95%CI)**	
		Crude[a]	Adjusted[b]	Crude[a]	Adjusted[b]	Crude[a]	Adjusted[b]
	**No.**	3,507	3,507	3,387	3,387	3498	3498
pooled sample 1, 2 and 3	**PFOS**	-43.8 (-75.3, -12.4)	-45.2 (-76.8, -13.6)	-26.4 (-56.2, 3.4)	-25.2 (-55.3, 4.8)	-0.05 (-0.11, 0.01)	-0.04 (-0.09, 0.02)
	**PFOA**	-90.1 (-117.9, -62.2)	-35.6 (-66.3, -5.0)	-78.5 (-104.8, -52.2)	-24.0 (-53.2, 5.2)	-0.17 (-0.22, -0.12)	-0.05 (-0.10, 0.01)
	**No.**	2,120	2,120	2,057	2,057	2,115	2,115
pooled sample 2 and 3	**PFHxS**	-59.3 (-88.3, -30.2)	1.2 (-28.3, 30.7)	-55.4 (-82.9, -28.0)	3.1 (-24.9, 31.2)	-0.10 (-0.15, -0.05)	0.02 (-0.04, 0.07)
	**PFNA**	-60.4 (-94.1, -26.7)	-36.3 (-70.6, -2.0)	-46.0 (-78.2, -13.8)	-20.6 (-53.7, 12.5)	-0.08 (-0.14, -0.02)	-0.02 (-0.08, 0.04)
	**PFHpS**	-64.1 (-97.1, -31.1)	-38.9 (-72.6, -5.1)	-57.8 (-89.3, -26.2)	-26.5 (-58.9, 6.0)	-0.09 (-0.15, -0.03)	-0.02 (-0.08, 0.04)
	**PFDA**	-18.6 (-53.5, 16.2)	-9.0 (-43.2, 25.2)	0.8 (-32.9, 34.5)	10.4 (-22.9, 43.6)	-0.01 (-0.07, 0.06)	0.02 (-0.05, 0.08)

[a] Adjusted for a study sample indicator. [b] Adjusted for a study sample indicator, infant sex, infant birth year, gestational week of blood draw, maternal age, parity, socio-occupational status, pre-pregnancy body mass index (BMI), smoking and alcohol intake during pregnancy. [c] Excluded births with missing gestational age and also infants born < week 25 or > week 43 to avoid unstable z-scores in extreme gestational weeks.

Table S8. Adjusted differences (β) and 95% confidence intervals (CI) for birth weight in grams and gestational age in days per doubling of prenatal PFASs levels mutually adjusting for different PFASs.

	Adjusted differences in birth weight (β and 95%CI) [a]		
	pooled sample 1, 2 and 3	pooled sample 2 and 3	
	Model 1 [b]	Model 2 [c]	Model 3 [d]
**No.**	3,507	2,120	2,120
**PFOS**	-38.11 (-82.09, 5.88)	-48.12 (-135.39, 39.15)	-55.53 (-145.59, 34.54)
**PFOA**	-9.94 (-52.63, 32.75)	44.20 (-13.42, 101.82)	49.56 (-8.74, 107.86)
**PFHxS**	N/A	N/A	24.95 (-10.14, 60.05)
**PFNA**	N/A	-25.02 (-68.51, 18.48)	-54.24 (-105.83, -2.65)
**PFHpS**	N/A	-14.51 (-88.38, 59.36)	-35.15 (-114.00, 43.70)
**PFDA**	N/A	N/A	47.98 (-0.56, 96.52)
	**Adjusted differences in gestational age (β and 95%CI)[a]**		
	**pooled sample 1, 2 and 3**	**pooled sample 2 and 3**	
	**Model 1 [b]**	**Model 2 [c]**	**Model 3 [d]**
**No.**	3,522	2,132	2,132
**PFOS**	-1.61 (-2.52, -0.71)	-0.37 (-2.12, 1.38)	-0.37 (-2.19, 1.44)
**PFOA**	0.73 (-0.15, 1.61)	1.37 (0.21, 2.53)	1.41 (0.24, 2.58)
**PFHxS**	N/A	N/A	0.47 (-0.23, 1.18)
**PFNA**	N/A	-0.59 (-1.47, 0.28)	-0.95 (-1.99, 0.08)
**PFHpS**	N/A	-1.39 (-2.88, 0.09)	-1.78 (-3.36, -0.19)
**PFDA**	N/A	N/A	0.56 (-0.41, 1.54)

[a] Adjusted for a study sample indicator, infant sex, infant birth year, gestational week of blood draw, maternal age, parity, socio-occupational status, pre-pregnancy body mass index (BMI), smoking and alcohol intake during pregnancy. [b] Additionally co-adjusted for PFOS and PFOA in the same model. [c] Additionally co-adjusted for PFOS, PFOA, PFNA PFHpS in the same model. [d] Additionally co-adjusted for PFOS, PFOA, PFHxS, PFNA, PFHpS and PFDA in the same model.

Table S9. Adjusted Odds Ratios (OR) and 95% confidence intervals(CI) for low birth weight and preterm birth according to different cutoff points and per doubling of prenatal PFASs levels.

		<2260 g (1st percentile)	<2500 g (~2nd percentile)	<2650 g (3rd percentile)	< 35 completed weeks	< 36 completed weeks	< 37 completed weeks
Adjusted OR [a] and 95%CI, pooled sample 1, sample 2 and 3	**No. Cases**	34	61	102	32	62	112
	**PFOS**	1.1 (0.7, 1.9)	1.3 (0.9, 2.0)	1.7 (1.2, 2.3)	1.2 (0.7, 2.1)	1.5 (0.9, 2.3)	1.5 (1.1, 2.2)
	**PFOA**	1.0 (0.6, 1.6)	1.0 (0.7, 1.5)	1.2 (0.9, 1.7)	1.1 (0.6, 2.0)	1.0 (0.6, 1.5)	1.1 (0.8, 1.5)
Adjusted OR [a] and 95%CI, pooled sample 2 and 3	**No. Cases**	21	37	61	19	34	59
	**PFHxS**	1.3 (0.8, 2.1)	1.2 (0.8, 1.7)	1.1 (0.8, 1.5)	1.0 (0.6, 1.7)	1.0 (0.6, 1.4)	1.1 (0.8, 1.5)
	**PFNA**	1.4 (0.8, 2.7)	1.5 (0.9, 2.4)	1.5 (1.0, 2.2)	2.2 (1.1, 4.7)	2.3 (1.2, 4.2)	1.4 (0.9, 2.1)
	**PFHpS**	0.8 (0.5, 1.3)	1.0 (0.6, 1.5)	1.3 (0.9, 1.8)	1.0 (0.5, 1.8)	1.6 (1.0, 2.7)	1.5 (1.0, 2.1)
	**PFDA**	1.5 (0.8, 2.6)	1.2 (0.8, 1.9)	1.2 (0.8, 1.8)	1.7 (0.9, 3.1)	2.0 (1.3, 3.2)	1.7 (1.2, 2.5)

[a] Adjusted for a study sample indicator, infant sex, infant birth year, gestational week of blood draw, maternal age, parity, socio-occupational status, pre-pregnancy body mass index (BMI), smoking and alcohol intake during pregnancy.
